# Therapy effect on hand function after home use of a wearable assistive soft-robotic glove supporting grip strength

**DOI:** 10.1371/journal.pone.0306713

**Published:** 2024-07-11

**Authors:** Anke I. R. Kottink, Corien D. M. Nikamp, Foskea P. Bos, Corry K. van der Sluis, Marieke van den Broek, Bram Onneweer, Janneke M. . Stolwijk-Swüste, Sander M. Brink, Nicoline B. M. Voet, Johan S. Rietman, Gerdienke B. Prange-Lasonder

**Affiliations:** 1 Roessingh Research and Development, Enschede, The Netherlands; 2 Department of Biomechanical Engineering, University of Twente, Enschede, The Netherlands; 3 Department of Biomedical Signals and Systems, University of Twente, Enschede, The Netherlands; 4 Reade, Center for Rehabilitation and Rheumatology, Amsterdam, The Netherlands; 5 Department of Rehabilitation Medicine, University Medical Center Groningen, University of Groningen, Groningen, The Netherlands; 6 Sint Maartenskliniek, Ubbergen, The Netherlands; 7 Rijndam Rehabilitation, Rotterdam, The Netherlands; 8 Department of Rehabilitation Medicine, Erasmus Medical Centre, Rotterdam, The Netherlands; 9 De Hoogstraat Rehabilitation, Utrecht, The Netherlands; 10 Centre of Excellence for Rehabilitation Medicine, Brain Centre Rudolf Magnus, University Medical Centre Utrecht, Utrecht, The Netherlands; 11 Department of Rehabilitation Medicine, Isala, Zwolle, The Netherlands; 12 Rehabilitation Centre Klimmendaal, Arnhem, The Netherlands; 13 Department of Rehabilitation, Donders Institute for Brain, Radboud University Medical Centre, Cognition and Behaviour, Nijmegen, The Netherlands; 14 Roessingh Centre for Rehabilitation, Enschede, The Netherlands; University of Illinois Urbana-Champaign, UNITED STATES OF AMERICA

## Abstract

**Background:**

Soft-robotic gloves with an assist-as-needed control have the ability to assist daily activities where needed, while stimulating active and highly functional movements within the user’s possibilities. Employment of hand activities with glove support might act as training for unsupported hand function.

**Objective:**

To evaluate the therapeutic effect of a grip-supporting soft-robotic glove as an assistive device at home during daily activities.

**Methods:**

This multicentre intervention trial consisted of 3 pre-assessments (averaged if steady state = PRE), one post-assessment (POST), and one follow-up assessment (FU). Participants with chronic hand function limitations were included. Participants used the Carbonhand glove during six weeks in their home environment on their most affected hand. They were free to choose which activities to use the glove with and for how long. The primary outcome measure was grip strength, secondary outcome measures were pinch strength, hand function and glove use time.

**Results:**

63 patients with limitations in hand function resulting from various disorders were included. Significant improvements (difference PRE-POST) were found for grip strength (+1.9 kg, CI 0.8 to 3.1; p = 0.002) and hand function, as measured by Jebson-Taylor Hand Function Test (-7.7 s, CI -13.4 to -1.9; p = 0.002) and Action Research Arm Test (+1.0 point, IQR 2.0; p≤0.001). Improvements persisted at FU. Pinch strength improved slightly in all fingers over six-week glove use, however these differences didn’t achieve significance. Participants used the soft-robotic glove for a total average of 33.0 hours (SD 35.3), equivalent to 330 min/week (SD 354) or 47 min/day (SD 51). No serious adverse events occurred.

**Conclusion:**

The present findings showed that six weeks use of a grip-supporting soft-robotic glove as an assistive device at home resulted in a therapeutic effect on unsupported grip strength and hand function. The glove use time also showed that this wearable, lightweight glove was able to assist participants with the performance of daily tasks for prolonged periods.

## Introduction

Loss of hand function can be caused by a variety of aetiologies, such as trauma, neurological diseases (stroke, spinal cord injury (SCI), cerebral palsy), or orthopaedic conditions (osteoarthritis, rheumatoid arthritis) [1–3]. Depending on the progressive or regressive nature of the limitations of the hand, hand strength and hand function can be maintained or even improved to a certain level of functioning through intensive exercise programs during inpatient or outpatient rehabilitation or community-based physical therapy. Physical exercise and muscle strength training were noted as evidence-based rehabilitation intervention among a number of neurological (e.g., stroke, SCI and neuropathies) and musculoskeletal (including fractures and arthritis) disorders in an overview based on Cochrane reviews [4]. In stroke for example, training interventions should consist of high-dose intensive (i.e. high frequency, long duration, many repetitions and large effort), active, task-specific training to promote arm/hand function [5]. Although much time and effort is spent during the (sub)acute rehabilitation phase to improve arm/hand function, a large proportion of patients still have an incomplete functional recovery of the upper limb after discharge from rehabilitation, for example in the chronic phase after stroke [3,6]. This limits the execution of normal everyday activities, such as eating and drinking, personal hygiene, and performing household activities or hobbies and makes them more dependent on help from their family, friends and the community. Furthermore, learned non-use of the hand in daily life resulting from decreased hand function might limit independence even more [7]. In those cases, it is important to provide suitable assistance and aids to support everyday life tasks, in addition to hand rehabilitation, but current methods have their limitations. Tools such as adapted cutlery, shower accessories or one-handed can openers can compensate for functional limitations, but these often prevent active involvement of the affected hand(s). In turn, this negatively affects any remaining hand capacity, amplifying learned non-use, and so on.

Recent technological developments enable a promising solution for the involvement of the affected hand, with the availability of advanced soft-robotic devices on the market. Such devices intended to be light-weight, wearable and comfortable to use in everyday environments to assist with daily tasks for prolonged periods [8–10]. A direct benefit of assistive robotic gloves has been demonstrated over recent years on strength and functional ability in multiple studies with SCI patients, stroke patients, children with neurological hand limitations and elderly with age-related hand limitations [11–20], together with a positive experience on usability and acceptability [14–20]. When these devices apply assist-as-needed control, a hybrid type of support becomes possible: assisting ADL where needed, while stimulating active and highly functional movements within the user’s possibilities. An example of such a wearable soft-robotic device is the Carbonhand (Bioservo Technologies AG; Kista, Sweden), a glove that supports grip by assisting finger flexion. Previous studies with this device found that participants rated usability and feasibility positively and increased their pinch strength when using the glove versus not using the glove [16,21]. When comparing this intervention in large groups of elderly people and stroke patients with self-perceived hand problems in ADL, using the glove either as an assistive device (aid) or as a training tool (therapy) at home, not only a direct supportive effect was found, but also a first indication of a therapeutic effect in the training group was found, and remarkably also in the assistive group [22]. However, the power of this pilot study was too low to conclude whether the found therapeutic effect was an actual effect. Even from the current literature, this conclusion cannot be confirmed. Although many different dynamic hand orthoses were developed within the last decade [23], no other clinical study reported on the therapeutic effect of another assistive device than Carbonhand after prolonged use in the home environment. Therefore, based on data from the previous pilot study [22], a powered clinical study was designed with the motivation to investigate whether long-term use of the assistive Carbonhand system stimulates active use of the affected hand, resulting in an improved hand function.

The aim of the present powered study was to investigate whether six weeks use of an assist-as-needed grip supporting soft robotic glove (Carbonhand) as an assistive device at home during activities of daily life (ADL) resulted in a therapeutic effect on grip strength, pinch strength and hand function in diverse diagnosis groups of patients with hand function limitations. In addition, the retention of a potential therapeutic effect four weeks after cessation of glove use was assessed. In accordance with the findings of our pilot study [22], we expected that the use of a grip-supporting soft robotic glove for multiple weeks during ADL at home will result in improved grip strength, pinch strength, and hand function abilities. Assuming that the glove supports the re-use of the affected hand, a sustained effect at follow-up was expected. In addition, to better understand which users have the most benefit from using the soft-robotic glove, relations of subject characteristics and glove use with changes in hand strength and hand function over time were assessed. Our hypothesis was that effects are modulated by impairment level at baseline, other characteristics such as age, gender and underlying diagnosis don’t have a strong influence on changes in hand strength and/or function after soft-robotic glove use. Additionally, it is expected that larger use time will relate to larger improvements.

## Methods

A detailed description of the protocol has been published previously [24]. Below a summary of the most important details is provided.

### Study design

**Fig 1:**
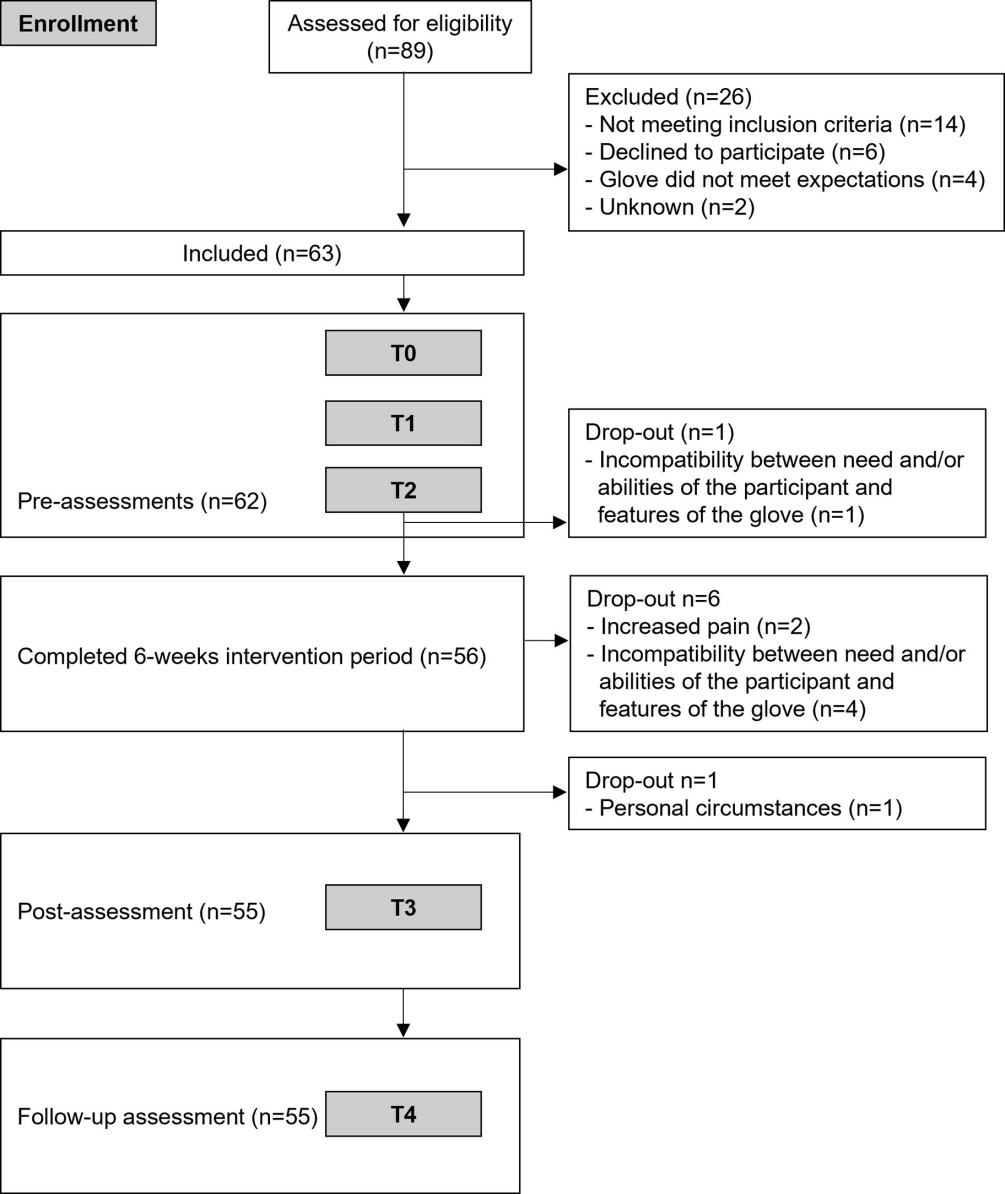
Study flowchart with overview of number of participants per assessment during the course of the study.

**Fig 2:**
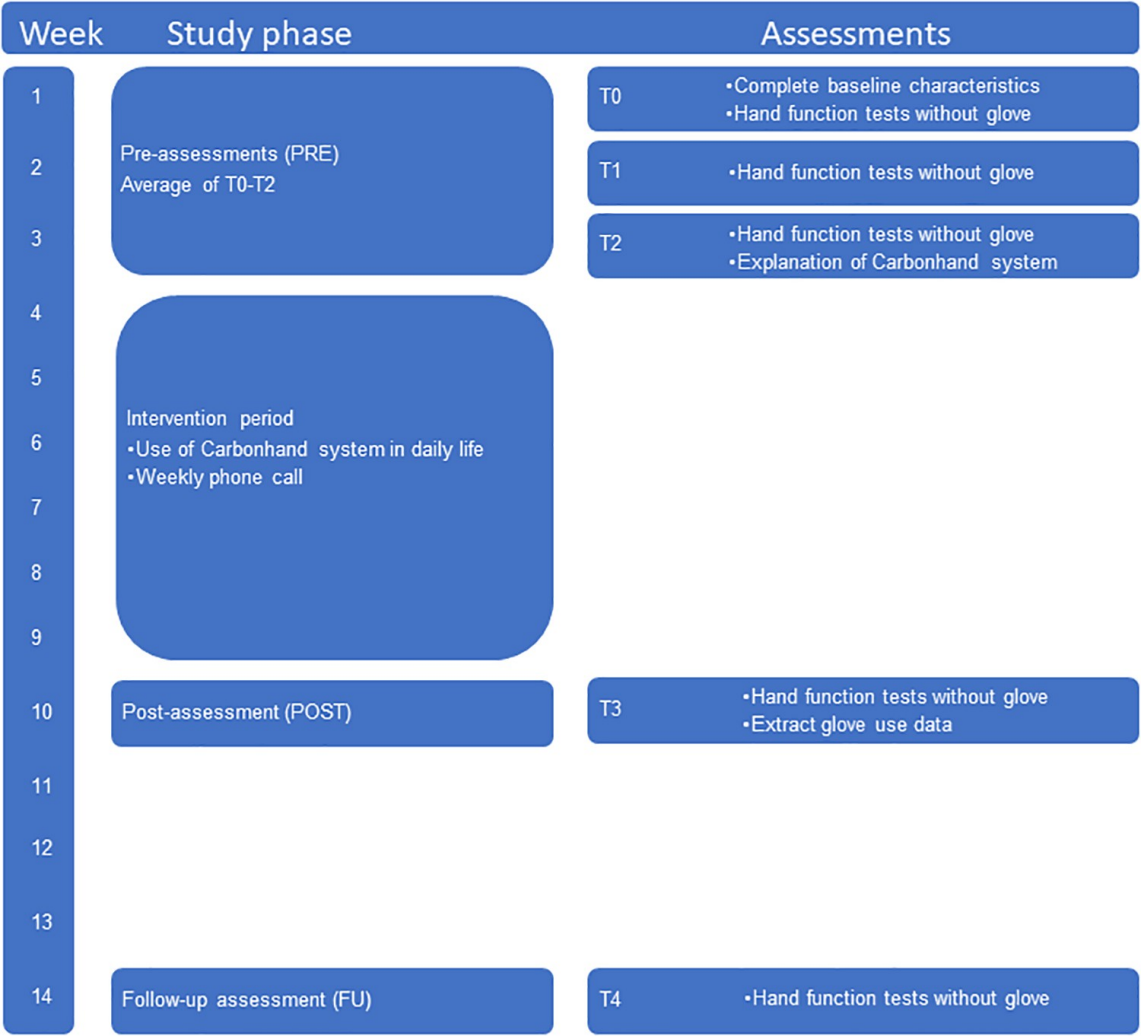
Overview assessments.

The study is an uncontrolled multicentre intervention trial (iHand). Fig 1 shows the TREND (Transparent Reporting of Evaluations with Non-randomised Designs) [25] study flowchart (S1 Checklist). Participants were assessed five times, including three pre-assessments (PRE), one post-assessment (POST) and one follow-up assessment (FU; Fig 2).

### Setting and study coordination

Eight clinical centres (rehabilitation centres and rehabilitation departments of (academic) hospitals) in the Netherlands participated in the study and collected all study data: Roessingh, Centre for Rehabilitation in Enschede, University Medical Center Groningen in Groningen, Isala in Zwolle, Rijndam Rehabilitation in Rotterdam, Reade in Amsterdam, De Hoogstraat Rehabilitation in Utrecht, Sint Maartenskliniek in Ubbergen and Klimmendaal in Arnhem. Roessingh Research and Development BV, Enschede, the Netherlands, coordinated the study and was responsible for study design, data management and data analysis. Clinical Trial Service BV, Losser, the Netherlands was contracted for external data monitoring according to Good Clinical Practice (ISO 14155:2020. Clinical investigation of medical devices for human subjects—Good Clinical Practice). Bioservo Technologies AB, Kista, Sweden is the manufacturer of the Carbonhand system and was the sponsor of the iHand study.

### Ethics

The Medical Ethical Committee of Twente (NL68135.044.19) approved the protocol and the study was registered in the Dutch national trial register (NL-OMON21637; https://onderzoekmetmensen.nl/en/trial/21637). In addition, the study was registered with the relevant Competent Authority (which was the Dutch Health and Youth Care Inspectorate at the start of the study, currently it is the Central Committee on Research Involving Human Subjects), since the study includes the use of medical device outside of its intended use. Recruitment took place from June 2019 till September 2022. Participants had to provide written informed consent before being screened and included into the study.

### Participants

Patients with chronic self-perceived hand function problems, including decreased handgrip strength, were enrolled by the participating clinical centres. The heterogeneous study sample included patients with acquired brain injury, osteoarthritis, rheumatoid arthritis, spinal cord injury, orthopaedic problems, and other neurological disorders. In case of two affected hands, the most affected hand was considered for inclusion in the study.

### Inclusion criteria

Inclusion criteria were (1) age between 18–90 years, (2) in a chronic and stable phase of disease, (3) previously received treatment for limitations in performing activities of daily living due to a decline in hand function (regardless of underlying disorder) at the involved rehabilitation centre or department, (4) capable of at least 10° of active extension of the wrist and fingers and 10° of active flexion of the fingers, (5) ability to make a pinch grip between thumb and middle or ring finger, (6) ability to put on the glove, (7) sufficient cognitive status to understand 2-step instructions (judged by personal contact between participant and experienced healthcare professional), (8) living at home, and (9) provided written informed consent. With regard to inclusion criteria 1, initially people could only participate when their age was between 18–80 years. However, since some centres had potential candidates with an older age in mind that could benefit from the glove, and who fulfilled to the remaining selection criteria, the Medical Ethical Committee agreed to increase the upper age limit.

### Exclusion criteria

Exclusion criteria were (1) severe sensory problems of the most affected hand, (2) severe acute pain of the most affected hand, (3) wounds on the most affected hand resulting in a problem when using the glove, (4) severe contractures limiting the passive range of motion, (5) comorbidities that limit functional use and performance of the arms and hands, (6) severe spasticity of the hand (≥2 points on Ashworth Scale), (7) participating in another study that can affect functional performance of the arm and hand, (8) receiving arm or hand function therapy during the course of the study, or (9) insufficient knowledge of the Dutch language to understand the purpose or methods of the study.

### Sample size

The sample size (power = 0.8; α = 0.05) was calculated at 56 participants, based on an expected mean improvement of 2.16 kg (SD 5.2 kg) [22]. Accounting for an expected 10% drop-out, 62 study participants need to be included. Since seven clinical centres initially participated [24], all aiming to include 9 participants, 63 study participants was the recruitment target.

### Carbonhand system

**Fig 3:**
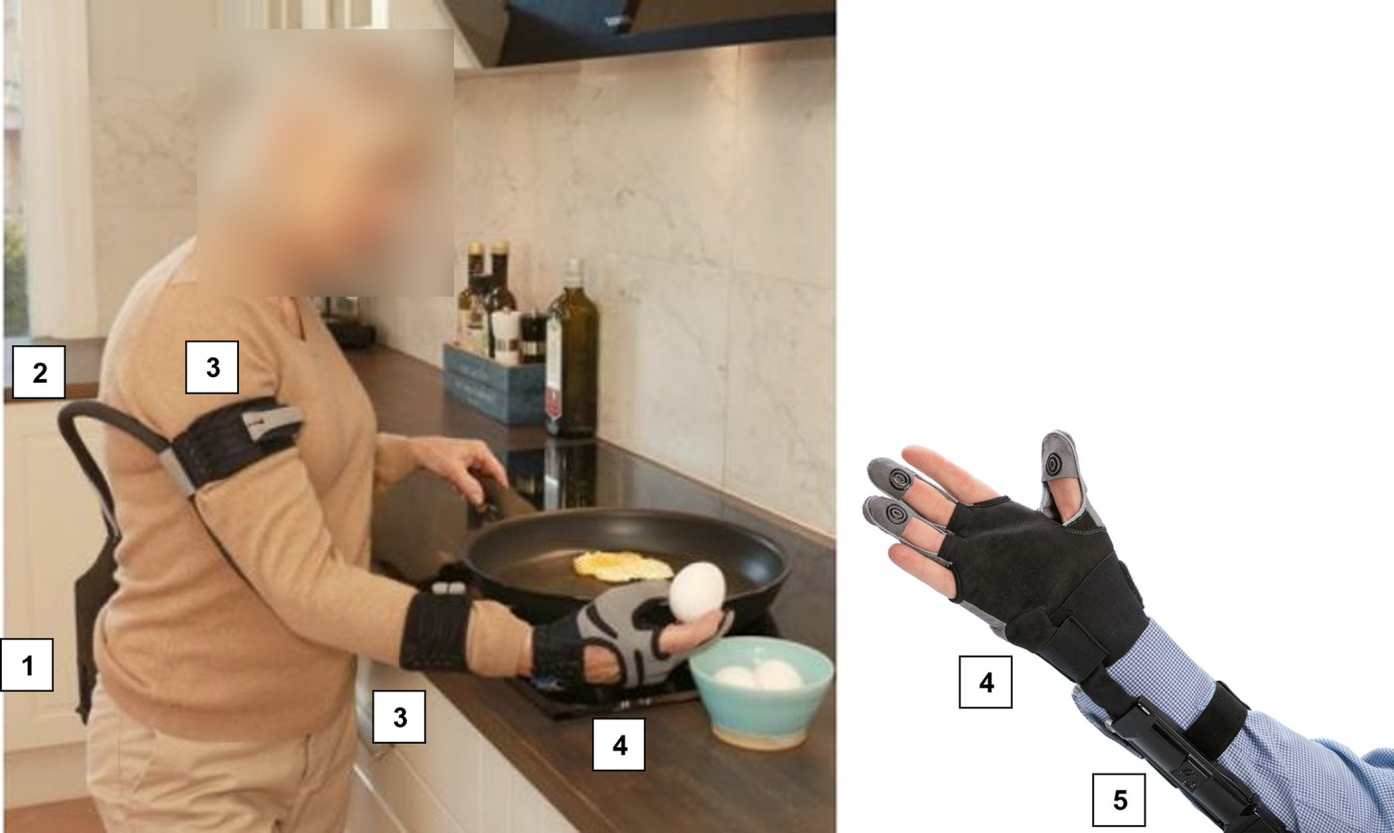
Overview of the Carbonhand system, consisting of a glove and a control unit to be used during activities of daily life. (1 = control unit, 2 = glove cord, 3 = arm straps, 4 = glove, with thumb, middle and ring finger supported, 5 = connection between glove and control unit; photos are property of Bioservo).

The soft robotic device Carbonhand is a system consisting of textiles and soft materials. Carbonhand is a CE-marked assistive medical device, but CE approval does not extend to the intended use in this study (therapeutic effect). The system consists of a glove and a control unit (Fig 3) and enhances a user’s grip based on voluntary, active initiation of the grip. Gloves are available in right- and left-hand versions and in several sizes (extra small–extra-large). The total weight of the system is approximately 700 grams.

### Glove

The purpose of the glove is to apply forces up to 20 Newtons per finger generated by the motors in the control unit and to provide the control unit with sensory input from touch sensors at the fingertips. Finger flexion is triggered by interaction forces between the fingertips and an object through pressure sensors at three fingertips (thumb, middle and ring finger) and actuates three separate motors to support flexion of those three fingers. Artificial tendons sewn into the glove along the length of the fingers apply the forces that induce finger flexion when contracted. The index finger and little finger are left uncovered allowing tactile sensing, while the thumb, middle and ring finger generate a power grasp.

### Control unit

The control unit is worn at the waist, on the hip or on the back of the user, using a clip or belt. The unit contains a battery, three motors and a microcontroller. A cable connects the control unit with the glove via a detachable connection close to the glove. Straps at the upper and lower arm, available in different sizes, lead the cable along the arm (Fig 3). The amount of assistive force is proportionally adjusted to help the user close the hand by embedded software in the control unit. An increase of force actively induced by the user and recorded at the sensors on the fingertips will increase the applied force by the actuators. Relaxation of the active grip reduces the interaction force, resulting in a gradual decline in force supporting finger flexion and allowing finger extension. The forces are calculated every 5th millisecond by the microprocessors in the control unit and adjust the tension in the artificial tendons to follow each movement of the user. The sensitivity and amount of force produced by the actuators was adjusted for each finger by the healthcare professional using a smartphone app. In addition, the configuration (which sensors activate which fingers) was set as well. Specific useful combinations of certain amount of sensitivity, force and configuration of finger activation could be saved as profiles under buttons of the control unit. These profiles were created for specific activities like carrying heavy objects or grasping small objects, or general purposes (low, medium, or high amount of support) to allow the user to switch per preference and need. Healthcare professionals created maximally three individual profiles in consultation with each user, depending on individual needs and situations.

### Study procedure

Extensive training sessions were organised to train all healthcare professionals involved in the study, including good clinical practice, the study protocol, fitting and operating the Carbonhand system and instructions to participants prior to the start of patient inclusion. A project website was used for easy access to all most recent project documentation.

Eligible candidates participated in a test session to explain the system and determine glove and strap sizes. During the session candidates could experience the support that the system can provide by testing the performance of several grasps for 10–15 minutes. After the session candidates decided whether or not to participate in the study.

Three pre-assessments (T0, T1, and T2) were scheduled across three weeks, one week apart, to assess baseline values for primary and secondary outcome measures (PRE). After the last pre-assessment, the correct glove size, finger length of the glove and maximally three profiles were individually determined by the healthcare professional. In addition, the participants were instructed how to use the system and practiced the use of the system together with the healthcare professional. Directly after the last pre-assessment an intervention period of six weeks started, in which the participants used the Carbonhand system for six weeks at home. During the period of home use, the healthcare professional had weekly phone contact to ask about the participants’ experience with the Carbonhand system, to verify adherence and to respond to any potential problems (adjust Carbonhand support profiles, device deficiencies, (serious) adverse events) that occurred. In case glove use was interrupted during the intervention period by unforeseen circumstances (like illness for few days or technical problems), participants were allowed to extend the period to achieve six weeks of glove use. Within one week after the end of the six week intervention period, a post assessment was conducted (T3, POST), followed by a follow-up assessment (T4, FU) four weeks later to measure the retention of effects.

### Intervention

Participants used the Carbonhand glove during six weeks in their home environment on their most affected hand. It was recommended to use the glove at least 180 minutes per week [26] during most common activities of daily living. Participants were free to choose for which activities, when and for how long the Carbonhand system was used. For safety reasons, participants were not allowed to use the Carbonhand system in combination with water without using a rubber (household) glove, in combination with hot objects without using an oven glove and while driving a bicycle, car, mobility scooter, wheelchair etc. A number of activities for which the glove might be supporting, were discussed between the participant and the healthcare professional.

### Outcome measures

#### Baseline characteristics

Baseline participant characteristics were collected by the healthcare professional during screening and at the first pre-assessment: age, gender (male, female, nonbinary), impairment or diagnosis, time since diagnosis, most affected side, dominant side, gloved side.

#### Primary and secondary outcome measures

Maximal hand grip strength, assessed with a dynamometer (Jamar hydraulic hand dynamometer, Patterson Medical) was determined as primary outcome measure. The average value over three attempts was used as the grip strength value. Secondary outcome measures were pinch strength of thumb with index, middle and ring fingers, and two measures of hand function: Jebsen Taylor Hand Function Test (JTHFT) and Action Research Arm Test (ARAT). All measures were performed without the glove on the most affected side and assessed during each assessment. In addition, cumulative use time was recorded automatically by the Carbonhand system during the intervention period. This reflects actual active use, because Carbonhand stopped recording use time after a period of two minutes without glove activation. The current work presents the effect of assistive glove use on objective measures of hand strength and hand function. An extensive overview of all outcome measures collected in the iHand trial can be found in the previously published protocol paper [24].

### Data management

A data management plan covered all aspects of handling data during and after the completion of the iHand project, both at the study coordinator’s site and at participating centres. More details can be found in the protocol paper [24]. A custom-designed case report form was built in a web-based clinical database (Castor, Castor EDC), which was used by the site investigators to collect all data using unique and anonymous participant codes. The monitor of the study visited all participating centers on average four-five times during the study to monitor the performance of the study protocol and collection of the data. Site investigators from each centre sent files with extracted glove use data (.csv) to the study coordinator by secured e-mail after completion of a dataset. After data collection was completed, the monitor checked the data collected on each participating centre and subsequently the study coordinator locked the clinical database to prevent alterations to the data and extracted all data for analysis.

### Statistical analysis

SPSS statistical software (version 19; IBM Corp.) was used to analyse the data. Normality was checked based on visual inspection via histograms and Q-Q plots, confirming with Kolmogorov-Smirnov tests where needed (p>0.05). In case of data deviating from a normal distribution, log transformations were applied to achieve normal distribution, where applicable, before analysing the intervention effects using parametric methods. If unsuccessful, non-parametric analyses were performed. Descriptive statistics were applied, presenting mean and standard deviation (SD), or median and interquartile range (IQR) in case of data deviating from a normal distribution, and minimum and maximum values for each outcome measure per session, and mean and 95% confidence intervals (CI), or median and IQR, of change scores between assessment intervals for each outcome measure.

Drop-outs were compared with those who completed the study for all baseline participant characteristics as well as cumulative use time, to better understand the demographic of drop-outs, using a Chi-square test (or Fisher’s exact test in case of frequencies <10) for ordinal variables (gender, dominant side, gloved side, diagnosis) and for continuous variables an independent t-test (age) or a Mann-Whitney U test (time since onset, grip strength). In addition, reasons for drop-out were noted, where available.

Data from the three PRE-assessments (T0/T1/T2) were visually inspected for patterns obviously deviating from random variation over time. In case of suspected patterns, paired-samples t-test (or Friedman test in case of data that deviated from a normal distribution) were performed to confirm whether values differed between T0, T1 and T2. This was only the case for JTHFT data, where values decreased consistently across sessions, with confirmed significant differences between each of the three PRE-assessments (p<0.001). In this case, the T2 value was used as PRE value for JTHFT to prevent overestimation of any potential intervention effect. In all other outcome measures, no pattern was obvious and T0, T1 and T2 values were averaged to represent the PRE intervention value.

To assess the effect of the intervention over time, linear mixed model repeated measurements analyses were chosen in the presence of missing values [27]. This was done for all outcome measures, of which the data had a normal distribution (i.e., grip strength, pinch strength of index, middle and ring fingers and JTHFT), with ‘session’ (PRE, POST, FU) as repeated factor (i.e., within-subjects factor). First, the best fitting covariance type was established per outcome measure by comparing -2 Log Likelihood scores against a *X*^*2*^ distribution. For each of these outcome measures, the best fitting covariance type, along with a model with ‘session’ (PRE, POST, FU) as fixed effect was used, which was ‘unstructured’ in all cases. Next, adding random effects and random intercepts was considered where possible to improve the fit of the model, however, this wasn’t applicable for any of the outcome measures. For data deviating from a normal distribution (i.e., ARAT), a Friedman test was performed to test differences between evaluation sessions. In case of significant differences, a Wilcoxon signed rank test was applied to discover between which sessions changes were significant, taking the Holm-Bonferroni correction for multiple testing into account.

Potential relations between change in the primary outcome measure and participant characteristics at baseline and use time were first explored by visual inspection of scatterplots or boxplots. In case of presumed (linear) relations, linear regression analysis was performed, entering the selected variables as independent variables in a regression model with changes in grip strength as dependent variable. This was done for both PRE-POST and PRE-FU differences in grip strength. In case of continuous variables, only those complying with a normal distribution were considered (i.e., PRE-POST changes in grip strength, PRE-FU changes in grip strength and age); log-transformed variables were used when this resolved deviation from a normal distribution (i.e., time since onset, baseline grip strength and use time). In case of discrete variables, dummy variables were created (coded 0 and 1 for each of the categories present, minus one that served as reference category) and entered into the regression analysis as independent variables (i.e., gender, affected side, dominant side, gloved side, diagnosis group). Normality and homoscedasticity of residuals was assessed post-analysis by inspection of histograms of residual values and scatterplots of standardized residuals against standardized predicted values, respectively. Regression analyses were only reported if they complied with above-mentioned assumptions and prerequisites. For all tests specified above, significance levels were set at α≤0.05.

## Results

### Participants

A total of 63 participants (Table 1 and S1 Data), between 18 and 79 years old, were included in the study, with limitations in hand strength and/or function mainly due to trauma-related injuries (incl. post-traumatic hand injuries, Chronic Regional Pain Syndrome) and neurological disorders (incl. stroke, spinal cord injury, multiple sclerosis, cerebral paresis), as well as neuromuscular disorders (incl. neuropathies and myopathies) and arthritis (rheumatoid arthritis, osteoarthritis). In the majority of participants (89%), the soft-robotic glove was worn on the dominant hand. In seven cases, the intervention period was extended with 1–2 weeks because of illness (n = 2), technical problems (n = 3), both illness and technical problems (n = 1) or personal circumstances (n = 1).

**Table 1:** Characteristics of participants at baseline.

Participant characteristics	All inclusions (N = 63)	Study completed (N = 55)	Drop-outs (N = 8)
Age (years)^a^	53.2 (SD 13.0) [18.0–79.0]	54.2 (SD 12.1) [25.0–79.0]	46.5 (SD 17.6) [18.0–63.0]
Gender ^b^ Male Female	29 (46) 34 (54)	26 (47) 29 (53)	3 (37) 5 (63)
Dominant hand^b^ Right Left	56 (89) 7 (11)	48 (87) 7 (13)	8 (100) 0 (0)
Gloved hand^b^ Dominant Non-dominant	48 (76) 15 (24)	40 (73) 15 (27)	8 (100) 0 (0)
Time since onset (years)^a^	8.4 (SD 13.2) [0.4–57.0]	9.1 (SD 14.0) [0.5–57.0]	4.3 (SD 3.5) [0.4–11.0]
Grip strength (kg)^a^	13.7 (SD 10.1) [1.1–48.9]	14.0 (SD 10.0) [1.1–48.9]	11.5 (SD 11.2) [1.3–37.8]
Diagnosis^b^ categorized as: Trauma-related injuries Neurological disorders Neuromuscular disorders Arthritis Other	27 (43) 13 (21) 11 (17) 9 (14) 3 (5)	27 (100) 8 (61) 9 (82) 8 (89) 3 (100)	0 (0) 5 (39) 2 (18) 1 (11) 0 (0)

^a^ Mean (SD) [min-max values].

^b^ Number of cases (% of total).

Eight of 63 participants dropped out during the intervention (Table 1), of which two had neuromuscular disorders, one arthritis and five neurological disorders. Reasons of drop-out were incompatibility between the needs and/or abilities of the participant and features of the glove system (n = 5), increase in experienced pain (n = 2) and personal circumstances (n = 1). Drop-outs weren’t different from those who completed the study in terms of age (p = 0.27), gender (p = 0.72), dominant side (p = 0.58), gloved side (p = 0.18), time since onset (p = 0.89) and PRE grip strength (p = 0.34). Drop-outs did differ in terms of underlying diagnosis (p = 0.014). Percentage of drop-outs was substantially higher among people with neurological disorders (n = 4 with cervical spinal cord injury and n = 1 with subarachnoid haemorrhage) compared to other diagnosis groups. Additionally, data is missing incidentally (noted in tables and figures where applicable), due to the inability to complete the test or due to Covid-19 related restrictions preventing physical visits to the test site. In the case of pinch strength tests, the inability to execute the test correctly (e.g., in case of a finger amputation or when the pinch grip was performed with support of other fingers) caused additional missing data.

No serious adverse events were reported during the study. A total of 46 adverse events (AE) occurred in 33 participants during the study. Of these, 32 AE involving 22 participants (70%) were considered by the reporting healthcare professional as possibly, probably or highly likely device-related. Reported AE regarded mainly pain and muscle ache (54%), as well as three to five AE reported for different issues, such as stiffness or tingling in hand/fingers, skin irritation under the glove and/or arm straps and illness and injuries/accidents unrelated to study participation. Among the eight drop-outs, four reported a total of six AE (pain and muscle ache (n = 3), stiffness in hand (n = 2) and tingling in fingers (n = 1)), which were all regarded as probably or highly likely device-related.

### Grip strength

**Fig 4:**
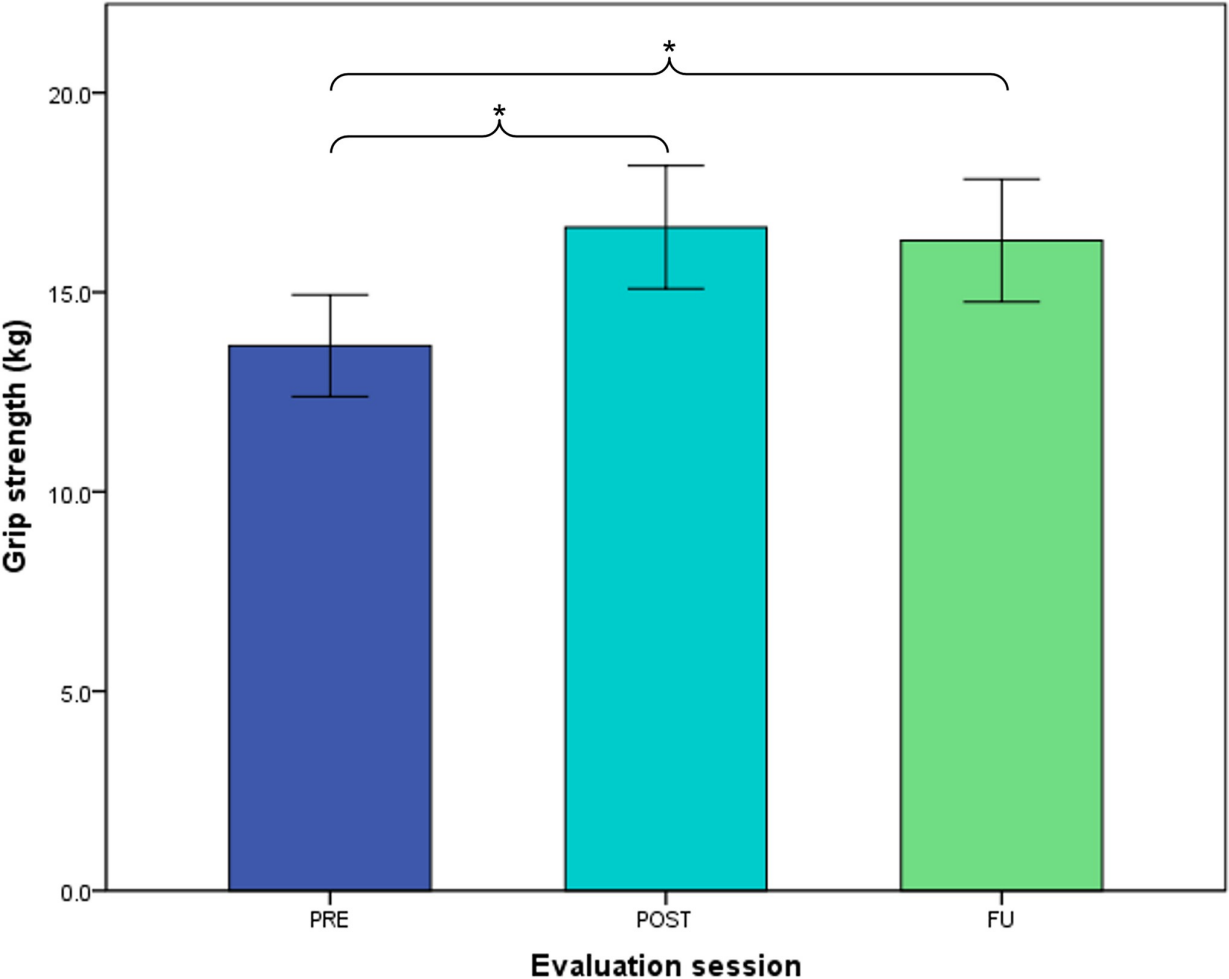
Average grip strength at PRE, POST and FU of six-week glove use. * indicating significant differences between sessions; error bars depicting standard error of mean.

Grip strength (Fig 4 and Table 2) improved over the six-week use period (main effect of ‘session’; p = 0.005), with significant differences between PRE and POST (+1.9 kg, CI 0.8 to 3.1; p = 0.002) and between PRE and FU (+1.7 kg, CI 0.5 to 2.8; p = 0.012). This is equivalent to an average gain of +24% (CI 10 to 38) with respect to individual PRE values of grip strength, which was sustained after cessation of glove use. Individual variation was large in both PRE grip strength and changes over time. Individual changes in grip strength between PRE and POST varied between -10.1 and +14.8 kg, which was reflected in relative changes varying between -83% and +225%.

**Table 2:** Hand strength and hand function at PRE, POST and FU, and change scores between PRE-POST and PRE-FU with statistics outcomes.

	Averages (SD) [min-max values] per session			Mean change (95%-CI) per interval		
Outcome measure	PRE	POST	FU	Effect of session^#^	Change PRE-POST	Change PRE-FU
**Grip strength (kg)**	13.7 (10.1) [1.1–48.39] *(n = 63)*	16.6 (11.2) [1.3–52.3] *(n = 53)*	16.3 (11.3) [1.0–53.7] *(n = 54)*	*p = 0*.*005*	1.9 (0.8–3.1) *p = 0*.*002*	1.7 (0.5–2.8) *p = 0*.*012*
**Pinch strength (kg)** • ***Index finger*** • ***Middle finger*** • ***Ring finger***	3.1 (2.3) [0.0–11.7] *(n = 58)* 2.5 (1.7) [0.0–8.1] *(n = 57)* 1.6 (1.7) [0.0–7.8] *(n = 58)*	3.3 (2.4) [0.0–9.7] *(n = 50)* 2.8 (1.9) [0.0–7.7] *(n = 48)* 1.9 (1.9) [0.0–6.3] *(n = 48)*	3.4 (2.7) [0.0–11.2] *(n = 48)* 3.4 (2.7) [0.0–8.7] *(n = 49)* 1.9 (1.7) [0.0–7.0] *(n = 49)*	*p = 0*.*31* *p = 0*.*058* *p = 0*.*068*	0.2 (-0.1–0.5) 0.3 (0.0–0.5) 0.2 (0.0–0.4)	0.3 (-0.2–0.8) 0.4 (0.0–0.7) 0.3 (0.0–0.6)
**JTHFT (s)**	116.1 (126.7) [34.1–669.6] *(n = 63)*	101.7 (106.0) [33.3–531.6] *(n = 53)*	99.8 (102.6) [34.8–538.4] *(n = 54)*	*p = 0*.*002*	-7.7 (-13.4 –-1.9) *p = 0*.*020*	-8.6 (-13.6 –-3.5) *p = 0*.*002*
**ARAT (points)** ^§^	54.0 (8.0) [5.0–57.0] *(n = 63)*	56.0 (5.5) [7.0–57.0] *(n = 53)*	56.0 (6.3) [7.0–57.0] *(n = 54)*	*P<0*.*001*	1.0 (2.0) [–10 – 8] *p<0*.*001*	1.0 (2.0) [–4 – 9] *p = 0*.*001*

SD = standard deviation, CI = confidence interval; kg = kilogram; s = seconds.

^§^ median (IQR) [min-max values] are reported and non-parametric testing was used.

^#^ test of effect of SESSION; if significant, post-hoc tests results are reported in columns of changes between PRE-POST and PRE-FU; if not significant, no further p-values were reported.

### Pinch strength

Pinch strength change data was available from between 47 and 50 participants per assessment and per finger involved in the pinch grip (Table 2). Pinch strength improved slightly over six-week glove use, predominantly in middle and ring fingers and appeared to be retained or even slightly increased at FU, however these differences didn’t achieve significance (main effects of ‘session’: index finger p = 0.31; middle finger p = 0.058; ring finger p = 0.068). Again, individual variation was large, with total changes between PRE and FU ranging from -4.1 to +7.3 kg (index finger), -2.1 to +4.3 kg (middle finger) and -2.1 to +3.8 kg (ring finger).

### Hand function

**Fig 5:**
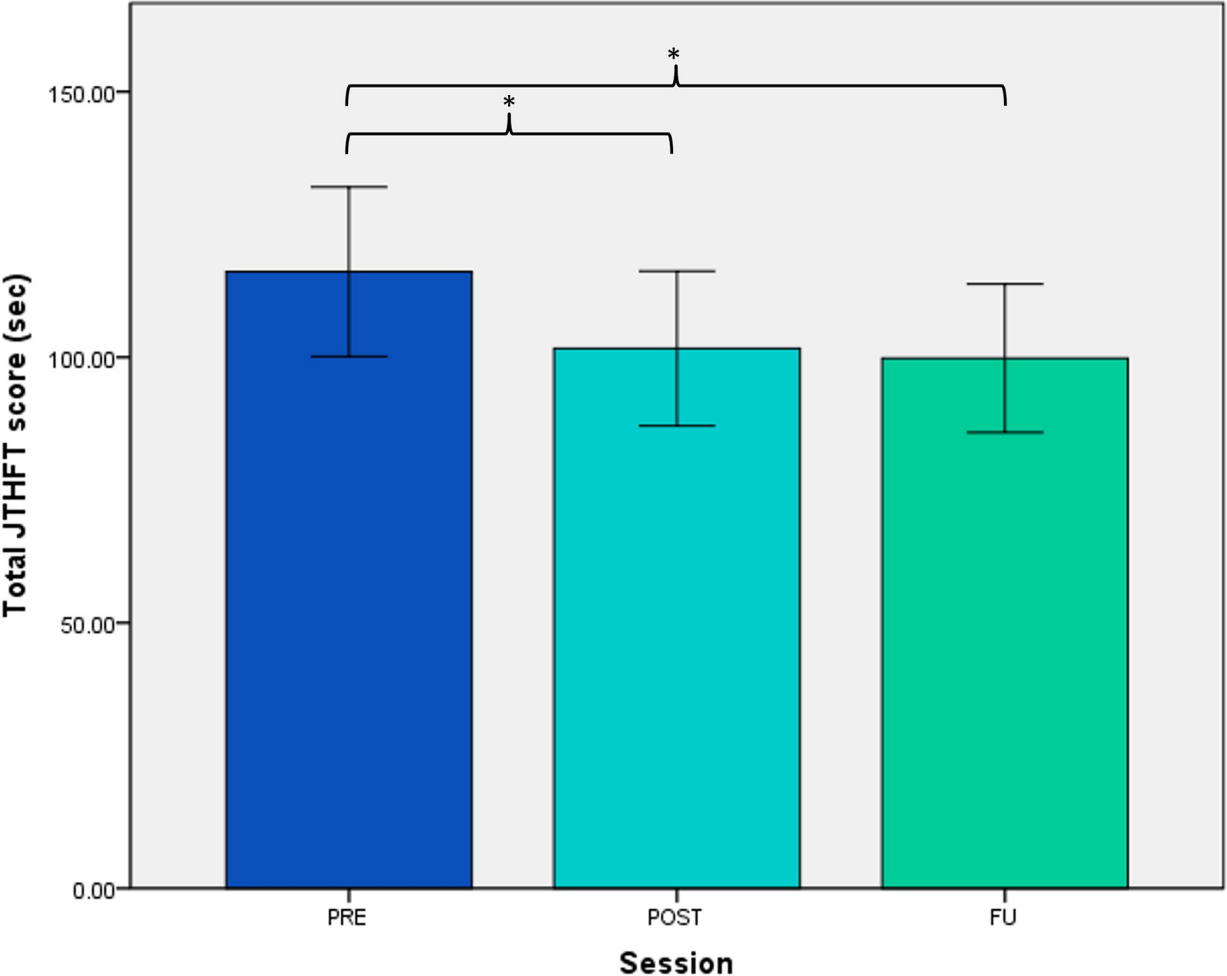
Average JTHFT score at PRE, POST and FU of six-week glove use. * indicating significant differences between sessions; error bars depicting standard error of mean.

Timed performance on JTHFT (Table 2) improved after six-week glove use (main effect of ‘session’, p = 0.002), with significant changes from PRE to POST (p = 0.020) and from PRE to FU (p = 0.002). Average change between PRE and POST was -7.7 s (CI -13.4 to -1.9) and between PRE and FU was -8.6 s (CI -13.6 to -3.5). Relative to PRE values, this presents average changes of +6% and +7% improvement, respectively. When looking at JTHFT scores per item (Fig 5), improvements were observed across all items.

Functional performance on ARAT improved significantly (Table 2), although by a small extent of +1.0 point (IQR 2.0, range -8.0–10) between PRE and POST and +1.0 point (IQR 2.0, range -4-9) between PRE and FU (p≤0.001).

### Glove use time

Participants (excluding drop-outs) used the soft-robotic glove for a total average of 33.0 hours (h) (SD 35.3), equivalent to a mean of 330 min/week (SD 354) or 47 min/day (SD 51). Glove use data was missing for one participant and incomplete for another patient (covering only the final three weeks of the use period), both due to technical errors with data-extraction from the glove. Cumulative use time varied greatly between participants, ranging from 24 min total use time to more than 186 h (equivalent to 1848 min/week).

### Explaining individual variations

Change in grip strength showed a lot of variation between participants, but inspection of scatterplots and boxplots revealed no clear relations between changes in grip strength PRE-POST and PRE-FU and age, gender, affected side, dominant side and gloved side. Visual inspection of scatterplots indicated modest relations of gains in grip strength with time since onset (higher gains for shorter times since onset), baseline grip strength (higher gains for lower baseline grip strength) and use time (higher gains with larger use times). Also, diagnosis group seemed to play a limited role based on visual observation. Regression analyses showed, however, that these potential relations weren’t strong or significant. Regression analysis of time since onset, baseline grip strength and use time resulted in non-significant models for both changes in grip strength PRE-POST (F(3,48) = 1.557, p = 0.21) and PRE-FU (F(3,49) = 1.425, p = 0.25), with an explained variance of respectively 9% and 8%. In case of diagnosis, modelled by four dummy variables to represent the five groups, no significant contribution was found for either PRE-POST (F(4,48) = 2.018, p = 0.11) or PRE-FU (F(4,49) = 1.936, p = 0.12) changes in grip strength, with an explained variance of 14% in both cases. Repeating the analyses with exclusion of three outliers, selected based on visual inspection of scatterplots, didn’t change the outcomes, supporting robustness of the finding of lack of explained variance by the current variables.

## Discussion

Assistive use of the Carbonhand soft-robotic glove in ADL had a positive therapeutic effect on grip strength and functional task performance. This study found an average increase in grip strength of 1.9 kg. Reported minimal clinically important differences (MCID) of grip strength varies with populations, e.g., 5.0 kg in stroke [28], 6.5 kg in people with radius fractures [29], and between 2.4 and 2.7 in healthy persons [30] and is not established yet for all populations included in the present study [31]. This suggests that the average increase in grip strength in the present study isn’t clinically meaningful, although the relative increase averaging at 24% indicates that grip strength improved by a quarter of their initial level. Considering the large variation between participants and the lack of MCID values for all involved populations, the changes experienced on individual level could reflect a relevant change, especially for people with low initial grip strength. The group average change in JTHFT of -7.7 s approaches MCID and about a third of the participants had improvements exceeding MCID [32], indicating that the therapeutic effect on task performance after glove use is clinically relevant. Improvements in grip strength and hand function occurred between PRE and POST assessment, and were retained four weeks after cessation of glove use. This indicates that changes seen during the use period can’t be contributed to only a direct assistive effect, but to increased unsupported movement ability as well. Moreover, this suggests that using an actuated assistive glove with grip support doesn’t cause a decline in arm/hand function over time.

### Therapeutic effect of assistive soft-robotic gloves

Comparing against existing literature, first of all the present findings confirm earlier pilot studies from our group, showing a therapeutic effect on unsupported JTHFT and pinch strength after four weeks assistive use of a predecessor of the soft-robotic glove in elderly with age-related limitations in hand function and in a small sample of stroke patients [20,22]. Moreover, the sustained improvements at four weeks follow-up found in the present study extends the findings from Radder et al., where only pre-post assessments were done [22]. Another research group found similar outcomes in SCI, with improvements in hand function after use of an earlier version of the same soft-robotic glove (SEM glove) as assistive device for 12 weeks, which were sustained at six weeks follow-up [33]. Strength of key and jaw grip also improved in the study of Osuagwu et al., while tip-to-tip pinch strength did not [33]. Those outcomes are also in line with the findings of the present study, where pinch strength was the only outcome measure that didn’t improve significantly. However, we didn’t measure key or jaw grip strength.

An extensive review from 2016 identified a total of 165 different dynamic hand orthoses, of which 12 were reported to have a dual purpose of assistance and therapy [23]. The listed dual purpose devices were all developed within the last decade. Accordingly, very few of the devices have been tested clinically [34]. Among 28 studies in a review on arm devices with both assistive and rehabilitation purposes [35], only two assessed the therapeutic effect of prolonged use of the assistive device in daily life. This concerned the previously mentioned studies [22,33]. To the best knowledge of the authors, no other studies have been published so far that allow for a direct comparison with the present study, applying a wearable soft-robotic glove as assistive device during prolonged use in ADL and assessing its therapeutic effect.

When considering the application of robotic hand devices as a training tool during supervised therapy sessions, multiple reviews, all focusing on stroke rehabilitation, have concluded that robot-assisted hand training has a superior effect on improvement of motor function, dexterity and spasticity [36–38] compared to conventional therapy. Those improvements persisted at one to 12 weeks follow-up [38]. Although those reviews included studies with non-robotic devices (e.g. sensorized gloves for biofeedback), non-wearable devices (e.g., semi-stationary work stations) and devices for the proximal arm as well, the general outcomes are similar to those of the present study. Interestingly, the present results after prolonged and unsupervised use of the soft-robotic glove as an assistive device are comparable to those after dedicated therapy sessions using a robotic hand device as a tool for supervised training. This suggests that unsupervised assistive home use might be as effective as using such a device during therapy sessions. However, a direct comparison against a control condition is required before this can be confirmed, which is highly relevant in context of current and future challenges to keep healthcare accessible and affordable.

### Potential role of use time

One of the factors that may be associated with the therapeutic effect of using a robotic assistive device is treatment dose. Average use time in the present study was 5.5h per week for six weeks (representing 47 min/day), which is higher compared to other studies evaluating the effect of training sessions with soft-robotic gloves: treatment dose ranged between 2.3 and 3.3 h per week for a training period of two weeks (i.e., representing 20–28 min/day) [39–41]. In the case of studies evaluating prolonged use of soft-robotic gloves as assistive devices in daily life, dose (i.e., use time) was similar as the present study with an average use time representing 45 min/day in Radder et al. [22] and considerably larger in Osuagwu et al. with 2.9 h/day in the first six weeks followed by 2.2 h/day in the final six weeks [33]. When directly comparing a group using a previous version of the soft-robotic glove as assistive device with a group using the same glove as a training tool at home connected to a computer-based game environment, use times were on average twice as high in the assistive group [22], indicating the potential of integrating training in activities of daily living.

Based on previous research [22,42] and in line with existing literature [26], we expected that longer use times were associated with larger improvements in hand function. In the present study however, no substantial relation between use time and changes in grip strength PRE-POST and PRE-FU was found. Likewise, another study showed no evidence of a dose-response effect, in terms of number of repetitions, for task-specific arm/hand training on functional capacity in chronic stroke patients [43]. Nevertheless, instead of a continuous relationship between use time and hand function gains, a more dichotomous association might be involved that could explain the observed lack of influence of use time in a continuous, linear way. In neurorehabilitation, it is thought that a particular level of functioning needs to be achieved before affected hand use is continued in daily life [44,45]. It is conceivable that the use of a soft-robotic glove as assistive device can enable people to use their affected hand more in daily life, or in additional activities that usually weren’t performed with the affected had, with ultimately improved hand strength and function as seen in the present study. Therefore, the exact role and potential mechanisms of use time in therapeutic effect soft-robotic glove use should be studied further.

### Identifying candidates for soft-robotic glove

For clinical practice it is important to gain insight into which users are likely to benefit most from the therapeutic effect of soft-robotic glove use. Based on the present findings, subject characteristics assessed in this study and the therapeutic benefit on grip strength of using a soft-robotic glove as assistive device don’t have a straightforward relation. A more complex interplay of various, and perhaps other, factors is likely involved. This currently prevents identification of subgroups prone to benefit from therapeutic effects of the glove. A first indication does come from a practical observation in the current study, with people in the neurological group displaying good outcomes, whereas drop-outs were relatively high among the neurological group. It seems that especially within this group, and more specifically SCI patients, it is very important to select the right users who will be able to use the glove properly and/or where the glove meets an existing need. This indicates that diagnosis group could play a role, in combination with other factors. Further analysis of subgroups would be beneficial to better understand which patients have to best chance for a good response to soft-robotic glove use.

### Study limitations and clinical implications

Some considerations of the present study are important for adequate interpretation of the results. The absence of a control group is an obvious limitation to interpret the effects, although the sample size was based on power calculations. In addition, the potential presence of a selection bias, including high proportions of motivated participants, needs to be taken into account.

All participants were included on basis of a self-perceived decline in hand function in daily life, however, we didn’t include a specific threshold to define declined hand function or grip strength. As a result, large variations in PRE values of grip strength were measured, with individual outliers up to 48.9 kg. High baseline levels in combination with potential day to day variation might explain individual negative effects found in the study and might blur the effects in patients with lower levels of grip strength. In addition, ARAT data showed clear ceiling effects, with a large proportion of participants scoring (near) maximal values, which hinders proper evaluation of changes in dexterity after six weeks glove use.

In addition to variability in amount of grip strength at baseline, aetiology of participants was heterogeneous since we decided to include patients based on a common symptom, which the glove addresses specifically. Consequently inclusion wasn’t limited to specific patient categories and underlying mechanisms of declined hand function are heterogeneous as well. Therefore, mechanisms of action of the soft-robotic glove are expected to vary as well, although the present findings don’t show a substantial role for diagnosis group as predominantly influencing the outcomes. Therefore, future studies on effects of robotic glove use in more homogeneous participants are advised to better understand the added value of these assistive devices for specific patients. On the other hand, participants had to have relatively good hand function to be able to don and doff the glove independently, to trigger the actuation of the glove and to induce release of the grip support. This might present a bias to the participants included in the study, preventing generalisation to the entire patient groups involved.

Despite these limitations, the present study did show that unsupervised use of a grip supporting soft-robotic glove as an assistive device for six weeks at home resulted in a therapeutic effect on grip strength and hand function. These improvements were generally sustained, or even improved, at follow-up. In addition, the observed glove use time showed that this wearable, lightweight glove was able to assist participants with the performance of daily tasks for prolonged periods. This implies that smart assistive devices can offer a hybrid type of support: assisting ADL where needed, while stimulating and motivating active and highly functional movements within the user’s possibilities. Essentially, soft-robotic gloves have potential for turning performance of everyday activities into highly intensive and task-specific training, while directly supporting and motivating patients to use their affected arm in functional activities. This could extend rehabilitation to people’s homes after inpatient or outpatient care and integrate rehabilitation in non-therapy hours during inpatient or outpatient care. Expanding available rehabilitation programmes with hybrid assistance-training paradigms via wearable robotic devices may contribute to sustainable future healthcare scenarios.

Further research is planned to investigate the underlying working mechanism of the therapeutic effect of the Carbonhand system. In a single case experimental design study it will be investigated if the Carbonhand system enables patients to use their affected hand more in ADL by the glove’s support than they did or could without using the system. Actual arm use will be tracked by wearing motion watch devices on both affected and non-affected arm on multiple days before participants start wearing the Carbonhand system, during the glove use period, and after cessation of glove use, in order to examine how assistive soft-robotic glove use influences actual use of the arm and hand in daily life (capacity) and its relation with changes in hand function (performance) and wellbeing.

## Supporting information

S1 Checklist Trend checklist. (PDF)

S1 Data SPSS file. (SAV)
